# A rare case of non-contiguous multifocal spinal tuberculosis

**DOI:** 10.11604/pamj.2017.26.157.9536

**Published:** 2017-03-16

**Authors:** Hilal Abboud, Ahmed Elhankari

**Affiliations:** 1 Neurosurgery Department, Med V, Souissi Medical School, Ibn Sina Hospital, Rabat, Morocco; 2 Radiology Department, Med V, Souissi Medical School, Ibn Sina Hospital, Rabat, Morocco

**Keywords:** Spondylodiscitis, non-contiguous, multifocal

## Image in medicine

Spinal tuberculosis is defined as an Infection of the intervertebral disc and the adjacent vertebral bodies, caused by Mycobacterium Tuberculosis (MT). It represents 40% of all osteo-articular spondylitis. It is a common disease in Africa, and recently there has been an upsurge in developed countries in relation to Human Immunodeficiency Virus infection. The early management is associated to a good outcome, and the diagnosis relies on imaging and disco-vertebral biopsy, it is characterized by a low amount MT in lesions and involves the slow multiplication MT. The treatment is based on antibacillary associated to surgical treatment in case of neurological deficit, and presence of vertebral column deformities. We report a rare case of a 46 years old patient, treated 3 years ago for pulmonary tuberculosis, who consults for diffuse back pain, the clinical examination finds a conscious patient, with a diffuse spinal syndrome, without neurological deficit or sphincter disorders. The radiological assessment shows a non contiguous multifocal spinal tuberculosis interesting the cervicodorsal and thoracolumbar junction.The patient underwent a vertebral biopsy, the histological examination confirms the spinal tuberculosis. He was proposed to surgical treatment to prevent the kyphosis progression.

**Figure 1:**
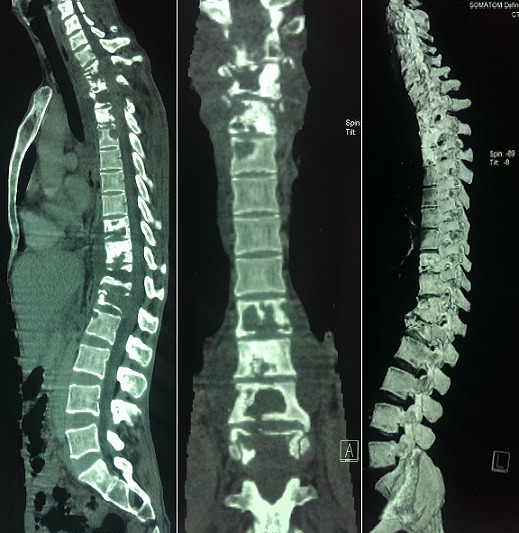
A) spinal CT scan on sagittal section (bone window) objective two non contiguous locations, cervicodorsal and thoracolumbar lesions with typical mirroring invasion of the vertebral plate; B) coronal section (bone window): note the normal aspect of the vertebral bodies between the two locations; C) 3D reconstruction: cervicodorsal (C7 to T4) and thoracolumbar (D10 to L1) achievement

